# Comparison of Sagittal Spinopelvic Alignment in Patients With Ankylosing Spondylitis and Thoracolumbar Fracture

**DOI:** 10.1097/MD.0000000000002585

**Published:** 2016-01-29

**Authors:** Tao Pan, Bang-Ping Qian, Yong Qiu

**Affiliations:** From the Medical School of Southeast University (TP); and Department of Spine Surgery, Drum Tower Hospital, Nanjing University Medical School, Nanjing, China (B-PQ, YQ).

## Abstract

This article is a comparative study. The aim of the study is to investigate the difference of sagittal alignment of the pelvis and spine between patients with thoracolumbar kyphosis secondary to ankylosing spondylitis (AS) and thoracolumbar fracture, and to evaluate the role of sacropelvic component in AS patients’ adaption to the changes in sagittal alignment.

Advanced stages of AS are often associated with thoracolumbar kyphosis, resulting in an abnormal spinopelvic balance and pelvic morphology, whereas thoracolumbar fractures may lead to major kyphosis with a potential compromise of the spinal canal, which can cause an abnormal spinopelvic balance. Until now, the comparison of that sagittal alignment between AS and thoracolumbar fracture is not found in the literature.

This study included 30 cases of AS and 30 cases of thoracolumbar fracture. Sagittal spinal and pelvic parameters were measured from the standing lateral radiograph, and the following 11 radiological parameters were measured, including global kyphosis (GK), thoracic kyphosis (TK), C7 tilt (C7T), sagittal vertical axis (SVA), spino-pelvic angle (SSA), lumbar lordosis (LL), upper arc of lumbar lordosis (ULL), lower arc of lumbar lordosis (LLL), pelvic incidence (PI), sacrum slope (SS), pelvic tilt (PT), and T9 tilt (T9T). Analysis of variance was used in the comparison of each dependent variable between the 2 cohorts. The relationship between sagittal spinal alignment and pelvic morphology of AS patients was determined via Pearson correlation coefficient (*r*).

Compared with the thoracolumbar fracture group, AS patients had significantly lower C7T, SSA, LL, LLL and SS (78.3° ± 9.3° vs 88.0° ± 2.7°, *P* < 0.001 for C7T; 91.6° ± 22.7° vs 119.1° ± 9.0°, *P* < 0.001 for SSA; 20.7° ± 21.0° vs 36.3° ± 16.8°, *P* = 0.001 for LL; 18.1° ± 11.9° vs 29.0° ± 9.7°, *P* < 0.001 for LLL; and 18.1° ± 11.9° vs 29.0° ± 9.7°, *P* < 0.001 for SS), whereas in terms of SVA and PT, AS patients had an obviously higher value than those of thoracolumbar fracture patients (94.5mm ± 58.4 mm vs 8.0mm ± 23.3 mm, *P* < 0.001 for SVA; and 26.5° ± 10.3° vs 17.5° ± 6.6°, *P* < 0.001 for PT). In AS patients, SS were found to be significantly correlated with SVA, SSA, and LL (*r* = −0.312, *P* < 0.05 for SVA; *r* = 0.475, *P* < 0.05 for SSA; *r* = 0.809, *P* < 0.001 for LL).

In our study, there were significant differences in sagittal alignment of the pelvis and spine between patients with AS and thoracolumbar fracture, and changes in pelvic morphology compensated more in AS patients for a thoracolumbar kyphosis. These findings may be helpful for better understanding of sagittal alignment in patients with thoracolumbar kyphosis secondary to AS.

## INTRODUCTION

A standing posture contains a delicate balance between the spine and pelvis.^1^ In order to minimize energy expenditure, a stable and compensated posture is obtained when these adjacent body segments are related and aligned closely.^2,3^ The sagittal balance is characterized by both pelvic and spinal parameters,^4^ and there has been an increasing recognition of the importance to evaluate the relationships between sagittal spinal and pelvic parameters.^1,2,5–9^ To date, some radiographical parameters are used to depict the spine, such as thoracic kyphosis (TK) and lumbar lordosis (LL).^10,11^ In addition, Legaye et al^12^ introduced 3 angles to assess the shape and orientation of the pelvis: pelvic tilt (PT), sacral slope (SS), and pelvic incidence (PI), due to the relation PI = PT + SS.

Ankylosing spondylitis (AS) is an inflammatory arthritis, which is characterized clinically by pain and stiffness of the back, and radiologically by arthritic changes in the sacroiliac joints and the entire spine.^13,14^ In the early stage of the disease, the sacroiliac joints are first involved,^15^ whereas advanced stages of AS are often characterized by a progressive stiffening of the spine and thorax.^16^ During the course of AS, the sagittal balance of the patient deteriorates, leading to a rigid thoracolumbar kyphosis.^17^ A severe thoracolumbar kyphotic deformity causes a downward tilt of the head and face and an anterior movement of the patient's trunkal center of mass.^18^ In order to compensate for the sagittal imbalance, the patient retroverts the pelvis positioning the hips in extension, flexes the ankles and the knees, and tilts the entire rigid segment of the spine backwards.^19^ Compared with flexion of the ankles and the knees, extension of the hips is much easier to be measured from the standing posterior-anterior and lateral radiographs of the entire spine, therefore becoming the mostly investigated parameter in the analysis of patients’ compensation in sagittal alignment.^20^ AS patients have an abnormal spinopelvic balance and pelvic morphology, but the role of sacropelvic component in their adaption to the changes in sagittal alignment is still under investigation.^17,19^ Thoracolumbar fractures may lead to major kyphosis with a potential compromise of the spinal canal, which can cause an abnormal spinopelvic balance. Until now, however, no published study has analyzed the spinopelvic morphology in AS patients with thoracolumbar kyphosis. Hence, the objectives of the present study were to investigate the difference of sagittal alignment of the pelvis and spine between patients with thoracolumbar kyphosis secondary to AS and thoracolumbar fracture, and to evaluate the role of sacropelvic component in AS patients’ adaption to the changes in sagittal alignment.

## SUBJECTS AND METHODS

### Subjects

A total of 30 patients with AS were included in this study from December 2008 to November 2012. The inclusion criteria were as follows: (1) patients had global kyphosis (GK)^21^ ranging from 40 to 120°; (2) no scoliosis or with a coronal curve <10°. Patients having previous spinal surgery, pseudarthorosis, discitis, or spinal fractures were excluded from the study. The diagnosis of AS was established by laboratory tests, radiographic features, and clinical features. There were 26 men and 4 women with an average age of 35.0 years (range, 19–62 years). Another thoracolumbar fracture group, including 11 men and 19 women, was also enrolled for comparison of sagittal spinopelvic alignment. The age ranged from 20 to 79 years and the average age was 51.7 years. The inclusion criteria were: (1) age ≥ 18 years, and with a thoracolumbar fracture > 3 months; (2) patients had a thoracolumbar kyphosis resulting from thoracolumbar fracture, with the apex located between T10 and L3; (3) no scoliosis or with a coronal curve <15°. Patients with leg length discrepancy of >1 cm were excluded. None of these people had a prior spine surgery, history of chronic back pain, or any neurological deficit. The study was approved by the Clinical Research Ethics Committee of our hospital.

### Radiological Assessment

**FIGURE 1:**
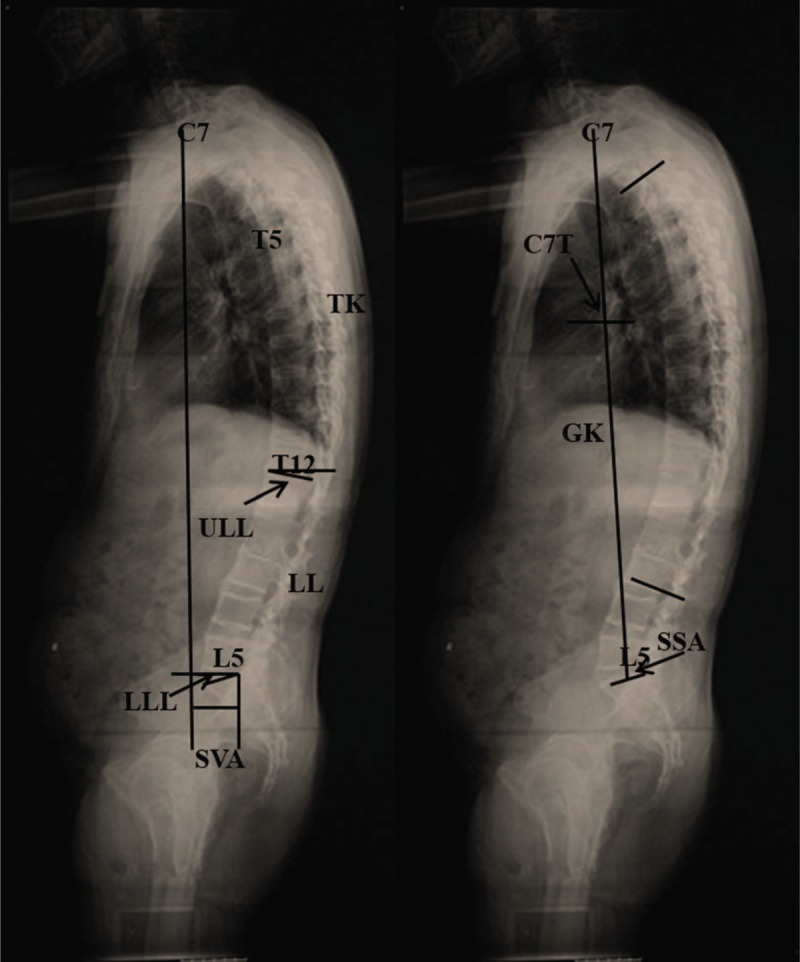
A 46-year-old man with ankylosing spondylitis. Sagittal spinal parameters were measured from the standing lateral radiograph. C7T is the angle between the horizontal plane and the line joining the center of C7 vertebral body and the center of the sacral endplate. SSA is the angle between the sacral endplate and the line joining the center of C7 vertebral body and the center of the sacral endplate. SVA is the distance between the C7 plumb line (C7PL) and the posterior superior corner of S1. GK is the Cobb angle between the upper endplate of the most tilted vertebra cranially and the lower endplate of the most tilted vertebra caudally. TK is the angle between the superior end plate of T5 and the inferior end plate of T12. LL is the angle between the superior end plate of L1 and the superior end plate of S1. ULL is the angle between the tangent line to the vertical axis at the apex of the lumber curve and the superior end plate of L1. LLL is the angle between the tangent to the vertical axis at the apex of the lumber curve and the superior end plate of S1. C7PL = C7 plumb line, C7T = C7 tilt, GK = global kyphosis, LL = lumbar lordosis, LLL = lower arc of lumbar lordosis, SSA = spino-pelvic angle, SVA = sagittal vertical axis, TK = thoracic kyphosis, ULL = upper arc of lumbar lordosis.

**FIGURE 2:**
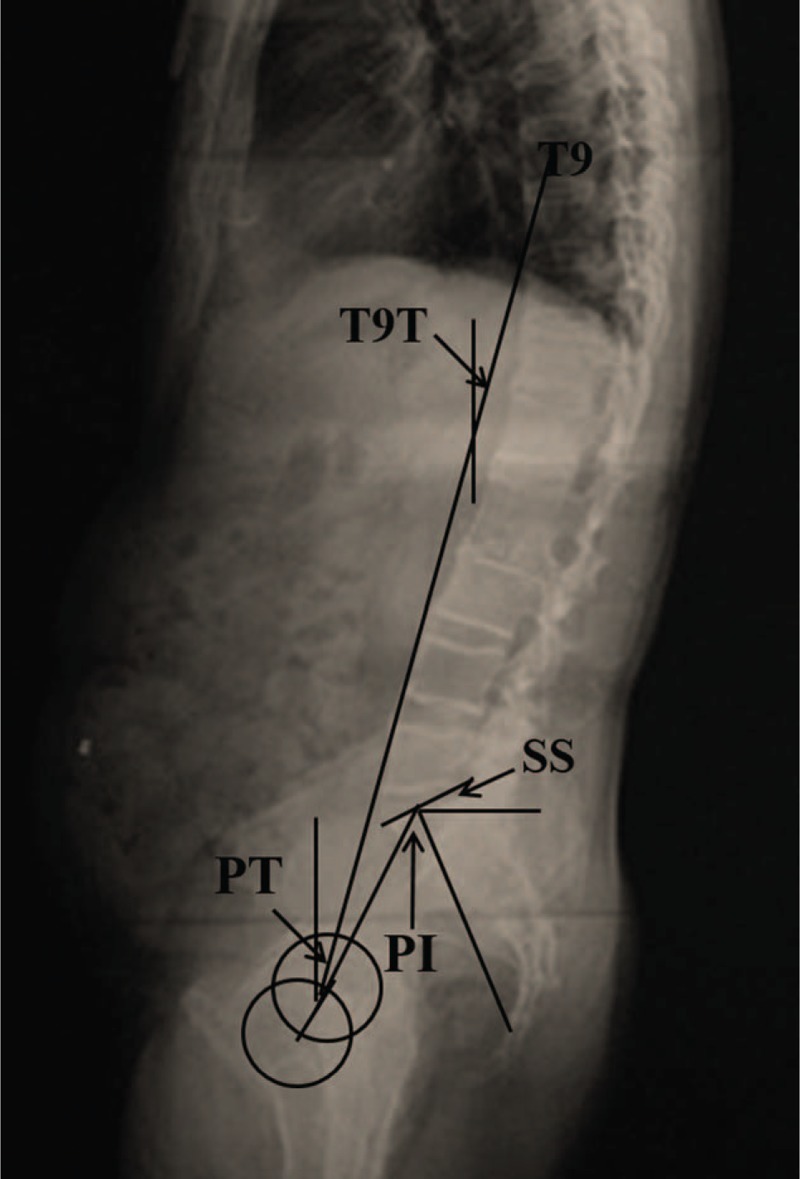
Sagittal pelvic parameters were measured from the standing lateral radiograph. PI is the angle between the line perpendicular to the sacral plate at its midpoint and the line connecting this point to the axis of the femoral heads. PT is the angle between the line connecting the midpoint of the sacral plate to the femoral head axis and the vertical axis. SS is the angle between the superior plate of S1 and a horizontal line. T9T is the angle between the vertical axis passing through the middle of both femoral heads’ centers and an axe passing through the center of T9 vertebral body. PI = pelvic incidence, PT = pelvic tilt, SS = sacrum slope, T9T = T9 tilt.

Standing posterior–anterior and lateral radiographs of the entire spine were obtained from patients with AS and thoracolumbar fracture in the fist-on-clavicle position.^22^ All the x-ray films were acquired in digital format. Using Surgimap (Spine Software, version 1.1.2, New York, NY), parameters related to sagittal spinopelvic alignment were then measured by the same spine surgeon. Duplicate measurements were taken for each parameter, and the average values were calculated. Measurements in the sagittal plane included (Figures 1 and 2):

1. C7 tilt (C7T):^16^ the angle between the horizontal plane and the line joining the center of C7 vertebral body and the center of the sacral endplate.
2. Spino-sacral angle (SSA):^16^ the angle between the sacral endplate and the line joining the center of C7 vertebral body and the center of the sacral endplate.
3. Sagittal vertical axis (SVA):^16,23,24^ distance between the C7 plumb line (C7PL) and the posterior superior corner of S1, positive when C7PL fell in front of S1 and negative when C7PL fell behind of S1.
4. Global kyphosis (GK):^21,24^ the Cobb angle between the superior endplate of the most tilted vertebra cranially and the inferior endplate of the most tilted vertebra caudally.
5. Thoracic kyphosis (TK):^23,25^ the angle between the upper end plate of T5 and the lower end plate of T12.
6. Lumbar lordosis (LL):^23,25^ the angle between the upper end plate of L1 and the lower end plate of S1.
7. Upper arc of the lumbar lordosis (ULL):^2,3,26^ the angle between the tangent line to the vertical axis at the apex of the lumber curve and the upper end plate of L1.
8. Lower arc of the lumbar lordosis (LLL)^2,3,26^: the angle between the tangent to the vertical axis at the apex of the lumber curve and the upper end plate of S1.
9. Pelvic incidence (PI)^11,27^: the angle between the line vertical to the sacral plate at its midpoint and the line linking this point with the axis of the femoral heads.
10. Pelvic tilt (PT)^27,28^: the angle between the line linking the midpoint of the sacral plate with the femoral head axis and the vertical axis.
11. Sacral slope (SS)^27,28^: the angle between the upper plate of S1 and a horizontal line. In geometry, LLL equals to SS.
12. T9 tilt (T9T)^11^: the angle between the vertical axis passing through the middle of both femoral heads’ centers and an axe passing through the center of T9 vertebral body.

### Statistical Analysis

Statistical analyses were performed using SPSS 13.0 statistical software (SPSS Inc, Chicago, IL). Statistical data are presented as mean ± SD. In the present study, an independent-samples *t* test was used in the comparison of 2 groups. The relationship between 2 variables was determined via Pearson correlation coefficient (*r*). Difference was regarded as significant when the *P* value was < 0.05.

## RESULTS

**FIGURE 3:**
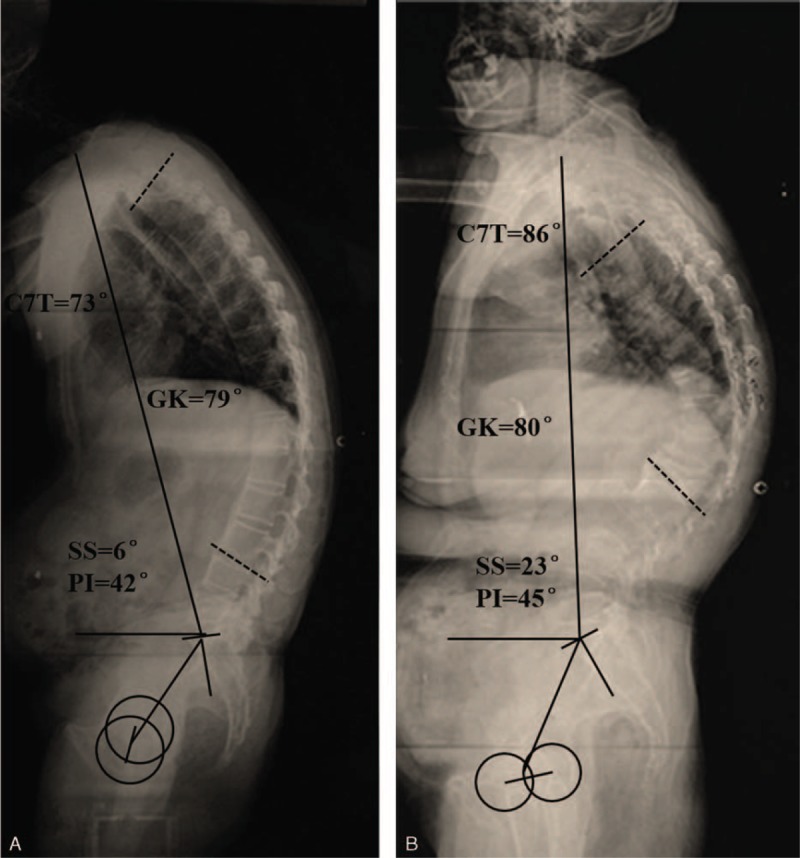
A comparison of sagittal spinopelvic alignment between AS patients (A) and thoracolumbar fracture patients (B). AS = ankylosing spondylitis.

The geometric parameters of sagittal spinal and pelvic alignment of the 2 groups were listed in Table 1. Compared with the thoracolumbar fracture group, AS patients had significantly lower C7T, SSA, LL, LLL, and SS (*P* < 0.001 for C7T; *P* < 0.001 for SSA; *P* = 0.001 for LL; *P* < 0.001 for LLL; and *P* < 0.001 for SS), whereas in terms of SVA and PT, AS patients had an obviously higher value than those of thoracolumbar fracture patients (*P* < 0.001 for SVA; and *P* < 0.001 for PT). However, no significant differences in GK, ULL, PI, or T9T were found between the 2 groups (*P* = 0.490 for GK; *P* = 0.406 for ULL; *P* = 0.323 for PI; and *P* = 0.069 for T9T). And Figure 3 showed the comparison of sagittal alignment between AS patient and thoracolumbar fracture patient.

**TABLE 1:**
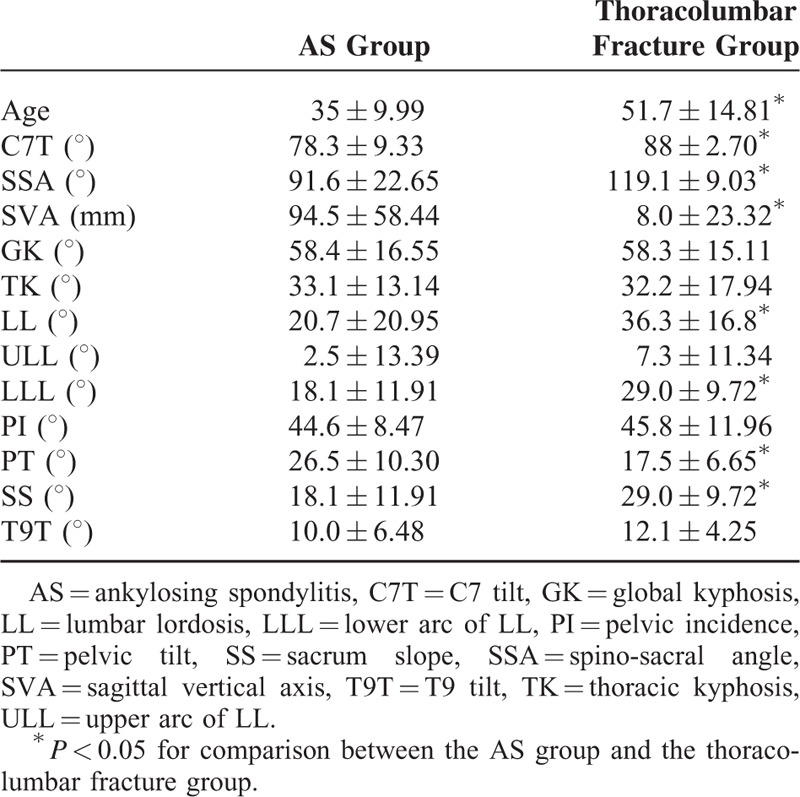
Comparison of Sagittal and Pelvic Parameters Between Different Groups

The correlation between sagittal spine parameters and the pelvic measurements for AS patients is shown in Table 2. SS was found to be significantly correlated with SVA, SSA, and LL (*P* < 0.05 for SVA; *P* < 0.05 for SSA; *P* < 0.001 for LL). PT was found to be significantly correlated with SSA and LL (*P* < 0.05 for SSA; *P* < 0.001 for LL). Also, PI was just observed to have significant correlation with LL (*P* < 0.05).

**TABLE 2:**
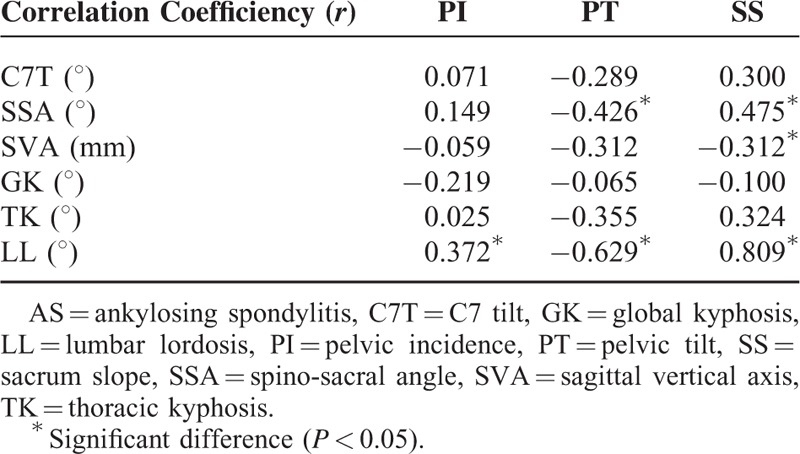
Correlation Coefficient (*r*) Between Sagittal Spine Parameters and Pelvic Measures for AS Patients

## DISCUSSION

Several studies have reported that the sagittal profile of the spine and pelvis influences the standing balance of healthy adult importantly.^5,7,8^ Berthonnaud et al^2^ proposed the concept of a linear chain linking the head to the pelvis to maintain a stable posture with minimum energy expenditure. Investigation of the spino-pelvic parameters provides for better understanding of the main compensatory mechanisms in patients with sagittal imbalance disorders.

It has been shown that patients with AS have an abnormal spinopelvic balance and pelvic morphology.^17,19^ During the course of AS, the thoracic kyphosis increased and the lumbar lordosis decreased, which dramatically restrict patients’ daily life, such as interpersonal communication and the activities of walking.^29^ In addition, the thoracolumbar kyphosis may cause significant sagittal imbalance. Debarge et al^16^ reported that compared with those of normal controls, AS patients’ SSA and C7T significantly decreased. Min et al^30^ observed a mean SVA value of 106.8 mm through a retrospective study of 11 AS patients. To the authors’ knowledge, no data have documented the spinopelvic morphology in AS patients with thoracolumbar kyphosis. Therefore, the aims of this study were to compare the sagittal lumbosacral spine morphology between patients with AS and thoracolumbar fracture, and to illustrate the role of sacropelvic component in AS patients’ adjust to the changes in sagittal alignment.

In the present study, both AS and thoracolumbar fracture patients had large global kyphosis. Nevertheless, in AS patients, SSA and C7T were significantly lower than those in thoracolumbar fracture patients; besides, AS patients had obviously higher SVA values than thoracolumbar fracture patients. Although PI was approaching in these 2 groups, AS patients had remarkably higher PT and lower SS when compared with thoracolumbar fracture patients, showing that different compensation patterns in terms of pelvic parameters might exist. The lumbar spine of AS patients could not compensate for increased thoracic kyphosis in the proximal region because of the loss of lumbar lordosis. Therefore, in order to compensate for the thoracolumbar kyphosis and to maintain a horizontal gaze, AS patients must depend more on pelvic retroversion through hip extension or ankle and the knee flexion.^19^ In the present study, the lumbar lordosis was much higher in thoracolumbar fracture patients (36.3° ± 16.8°) than in AS patients (20.7° ± 20.95°). From this study, we can see that thoracolumbar fracture patients’ pelvis and hips may not work in compensation for the localized thoracolumbar kyphosis, as they could have subsequent increase in lumbar lordosis to compensate for the localized thoracolumar kyphosis to achieve spinal balance, which is different from AS patients.

Compared with thoracolumbar fracture patients, AS patients had significantly higher SVA values and lower SS values. The Pearson correlation coefficient showed that SS was the only parameter significantly correlated with SVA, indicating that AS patients with lower SS could be more potential to have sagittal imbalance. Also, we can see that PT and SS were correlated with SSA and LL in this study. Several previous studies had reported that the SS angle is an essential component of overall sagittal alignment,^31^ which can be predictive of patients’ ability to compensate the sagittal imbalance. Pelvic tilting is the first way of compensation when the kyphosis occurs on a rigid spine of AS patient,^17^ which decreases the SS and increases the horizontal length between the femoral heads and the sacral plate.

To the best of our knowledge, this is the first study to systematically investigate the difference of sagittal lumbosacral spine morphology between patients with thoracolumbar kyphosis secondary to AS and thoracolumbar fracture. However, 1 limitation of the present study is that the ages were not matched perfectly between AS patients and thoracolumbar fracture patients.

In conclusion, this study demonstrated that there were significant differences in sagittal alignment of the pelvis and spine between patients with AS and thoracolumbar fracture, and changes in pelvic morphology compensated more in AS patients for a thoracolumbar kyphosis. In addition, we can see that AS patients with lower SS could be more likely to have sagittal imbalance. These findings may be helpful for better understanding of sagittal alignment in patients with thoracolumbar kyphosis secondary to AS.
